# Building evidence on what works (and what does not): practical guidance from the World Health Organization on post-project evaluation of adolescent sexual and reproductive health projects

**DOI:** 10.1093/heapol/czab028

**Published:** 2021-03-29

**Authors:** Susan Igras, Marina Plesons, Venkatraman Chandra-Mouli

**Affiliations:** Georgetown University’s Institute for Reproductive Health, Center for Child and Human Development, 3300 Whitehaven Road NW, Washington, DC 20007, USA; Department of Sexual and Reproductive Health and Research, World Health Organization/Human Reproduction Programme; Department of Sexual and Reproductive Health and Research, World Health Organization/Human Reproduction Programme

**Keywords:** Post-project evaluation, ex-post evaluation, post-hoc evaluation, lower- and middle-income countries, implementation guidance

## Abstract

Over the past 25 years, there has been significant progress in increasing the recognition of, resources for, and action on adolescent health, and adolescent sexual and reproductive health (ASRH) in particular. As with numerous other health areas, however, many of the projects that aim to improve ASRH are implemented without well-thought-out plans for evaluation. As a result, the lessons that projects learn as they encounter and address policy and programmatic challenges are often not extracted and placed in the public arena. In such cases, post-project evaluation (PPE) offers the possibility to generate learnings about what works (and does not work), to complement prospective studies of new or follow-on projects. To fill the gap in the literature and guidance on PPE, the World Health Organization developed *The project has ended*, *but we can still learn from it! Practical guidance for conducting post-project evaluations of adolescent sexual and reproductive health projects*. This article provides an overview of the guidance by outlining key methodological and contextual challenges in conducting PPE, as well as illustrative solutions for responding to them.

**KEY MESSAGES**

- There is a significant lack of evaluation planned as part of the project cycle, limiting the ability to demonstrate what was achieved and what approaches worked and did not work. While rigorous program evaluation is the ideal, post-project evaluation provides an untapped opportunity to generate learning to improve the future implementation of health and development projects, including adolescent sexual and reproductive health projects.
- The time lag between a project ending and conducting a post-project evaluation creates new opportunities to learn about programming, including real-time assessment of the sustainability of project efforts and the identification of unexpected and emerging outcomes. Such effects are not possible to evaluate at the moment that a project ends.
- Equally applicable to other sectoral projects in the health and development sphere, the WHO publication, *The project has ended, but we can still learn from it! Practical guidance for conducting post-project evaluations of adolescent sexual and reproductive health projects* is based on evaluators' technical expertise and practical experiences in conducting post-project evaluation*.* The Guidance contributes to bridging the ‘how-to’ gap.

## Introduction

Over the past 25 years, there has been significant progress in increasing the recognition of, resources for, and action on adolescent health, and adolescent sexual and reproductive health (ASRH) in particular (Chandra-Mouli *et al.*, 2015, 2019). Many countries have established policies and programmes that aim to improve ASRH, including some that are multi-sectoral with interventions related to health, education (including sexuality education), economic security and social/legal protection. Non-governmental organizations (NGOs) and civil society organizations (CSOs), including some that are youth-led, play a critical role in complementing government-led action through advocacy and service delivery.

As with numerous other health areas, many of the projects that aim to improve ASRH are implemented without well-thought-out plans for evaluation. A range of reasons helps to explain this phenomenon, including limited training and understanding of the value of evaluation, lack of dedicated evaluation resources, weak data systems and hesitation in a politicized environment to conduct evaluations (Morra-Imas and Rist, 2009; World Bank, 2013). As a result, at the end of a project, baselines and endlines may not exist, identifying a post-project comparison area can be complicated and monitoring data may not provide sufficient information to draw out lessons learned, especially related to the quality of the delivery of the intervention. However, many projects encounter and address policy and programmatic challenges, and in doing so, learn valuable lessons. Since careful documentation and rigorous evaluation are the exceptions rather than the norm, the lessons from these efforts are often not extracted and placed in the public arena.

In such cases, post-project evaluation (PPE) offers the possibility to assess the impact and generate learnings about what works (and does not work), to complement prospective studies of new or follow-on projects. While there is no standard definition for PPE, the Japan International Cooperation Agency (JICA, 2004) offers an operational definition which describes what this type of evaluation is, what its objectives are and how it is done:

Post-project evaluations are performed within a certain period after a project is completed, and most are not planned while projects are still operating. As staff may no longer be available, and project activities are not observable, PPE is based on existing reports, monitoring reports, other written information, and often include additional data collection. While such evaluations may assess the extent that projects implemented planned activities and how well outcomes were achieved, given they occur after projects end, such evaluation may also focus on long term impact and sustainability. PPEs consider retrospectively circumstances surrounding a project and other elements that may have influenced the implementation, impact, and sustainability of results.

Despite the potential they hold, there is a lack of literature on PPE, and technical guidance for conducting them is rare. A review commissioned by the World Health Organization (WHO) in 2018 found that while many institutional funding and technical assistance agencies have guidance for designing and executing formative, mid-term and final project evaluations, little attention is accorded to PPE. In particular, the review identified only six ASRH PPEs in seven evaluation clearinghouse databases. This lack of reporting was affirmed by another scoping study which noted that ‘fewer than 1% of all international development projects were evaluated after they ended’ (Zivetz *et al.*, 2017).

To fill this gap, the WHO developed *The project has ended, but we can still learn from it! Practical guidance for conducting post-project evaluations of adolescent sexual and reproductive health projects* to advance thinking on the utility and practical means of conducting PPE of ASRH projects (WHO, 2019). The WHO Guidance is equally applicable to other health and development projects, as well as to final project evaluations when baseline and other documentation do not exist or are insufficient, or when project aims have shifted mid-way through a project lifecycle. This article provides an overview of the WHO Guidance by outlining key methodological and contextual challenges in conducting PPE, as well as some illustrative solutions for responding to them.

## Methodology

The WHO Guidance is the result of several inputs. First, a review was conducted of published information from peer-reviewed and grey literature that included PPEs on ASRH projects posted between 2010 and 2017 in seven evaluation clearinghouses operated by institutional and multi-lateral organizations. The review identified critical challenges and crucial decision-points faced by evaluators working on PPE of ASRH projects, including several specific to adolescent programmes such as ensuring adequate consent and the protection of minors, whether they are respondents or members of an evaluation team. The identified methodological and contextual challenges and approaches to addressing them form the core of the WHO Guidance. Second, a scoping review was conducted of evaluation guidance from funding and technical assistance agencies working in low- and middle-income countries to identify existing guidance documents on PPE. Finally, technical consultation with seasoned evaluators and researchers was used to review the first draft of the WHO Guidance and to allow for critiques, clarifications and inputs of challenges and solutions from their own PPE experiences. These inputs led to revisions and the final document.

## Key post-project evaluation challenges and illustrative solutions

The review indicated that challenges exist throughout an evaluation, from planning through data collection to analysis and interpretation of findings. During evaluation planning, issues often arise, such as defining measurable outcome indicators that were not well defined by the project and establishing a PPE team that brings in historical perspectives and implementation knowledge. When conducting PPE with limited project data and other documentation, evaluators are challenged to re-purpose data or to collect new information from former participants, implementers and other stakeholders in rigorous-yet-feasible ways. In the absence of real-time data, evaluators need to determine how to identify and consider the external context—e.g. other projects, new policies and programmes or new environmental conditions—that may have influenced implementation and outcome achievements. Evaluations often involve debriefs with staff and other stakeholders to aid in the interpretation of findings. However, in the post-project context, this is not always possible to do, as the right people need to found and must be willing to contribute. Further, while PPE is well suited to assess sustainability and emerging and unanticipated outcomes, PPE evaluators may need first to define retrospectively what effects were supposed to be sustained at individual, interpersonal, community, institutional and other levels. This requires open-ended questioning to explore the unexpected.

In response, the WHO Guidance offers a range of field-tested strategies that can be used to mitigate or manage such challenges. Further, it provides case examples to illustrate selected strategies in action. Finally, it includes links to reference documents, including a draft evaluability assessment tool to guide evaluators in thinking through issues as they begin a PPE.

Below, we highlight challenges in the areas of preparing for an evaluation, managing lack of data availability and rigour and assessing context, complexity and sustainability effects. Several case studies from the WHO Guidance highlight these dilemmas and possible solutions. Readers are referred to the WHO Guidance for a detailed discussion of all of the identified challenge areas and illustrative solutions via case studies and discussions of realistic approaches from actual PPEs.

### Evaluation preparation

Because PPEs are not planned when projects are still operating, preparing for an evaluation in a post-project space has challenges. An overarching issue is determining a feasible evaluation design given the available time and resources, as well as challenges in finding past participants. Additionally, there is the challenge of managing stakeholder and evaluator expectations of possible PPE designs. An evaluator may need to select underused-yet-strong evaluation designs, such as endline-only comparison group designs, to provide a way forward. Furthermore, to achieve the evaluation aim, the evaluator may need to deviate from the expected approach and to identify and justify a different design.

The review identified other common dilemmas such as:

- having an insufficient definition of a project's change theory or outcome parameters to guide the evaluation;
- assembling an evaluation team with sufficient historical and contextual understanding of the past project's implementation and context; and
- avoiding recall and other biases when bringing past implementers and participants into a PPE team.

The two case examples below highlight dilemmas and feasible preparation strategies for PPEs.

Using a new evaluation design when the original one is not feasible: a project in Sierra Leone

Recognizing that in Sierra Leone adolescent pregnancy is significantly driven by sociocultural factors, UNICEF established a partnership with Child Fund, Save the Children, the Council of Churches of Sierra Leone, Restless Development and the Bangladesh Rural Advancement Committee. Collectively, these organizations designed and implemented five pilot projects in seven districts intending to address underlying drivers of adolescent pregnancy in the country (2010–12). They subsequently sought to conduct a post-project evaluation to foster learning about the actors, contexts and challenges of preventing/reducing adolescent pregnancy to inform future programming.

The plan was to use a pre-/post-intervention evaluation design to assess impact, given that each partner had conducted baseline studies. However, this plan was not possible due to the differences in the interventions and measurement approaches used by each pilot project. Instead, the evaluators selected an outcome mapping evaluation approach to assess, across the five projects, the different dimensions of change in mindsets, contexts, attitudes and perceptions of individuals and groups that influence adolescents’ motivations, interactions, relationships and behaviour. It also explored the contributions of the pilot projects in supporting girls’ empowerment and increasing their access to and use of sexual and reproductive health services.

*Source*: Farzaneh (2013)

Establishing a shared understanding of a past project: CERCA in the Plurinational State of Bolivia, Ecuador and Nicaragua

The Community-Embedded Reproductive Health Care for Adolescents (CERCA) Project was implemented in Bolivia, Ecuador and Nicaragua (2011–14) to test the effectiveness of contextually adapted interventions to prevent teenage pregnancies. Across countries, CERCA worked at multiple levels—engaging adolescents, their peers, parents and health care providers.

A mixed-method post-project evaluation was planned to assess the process and impact of the project, examining how CERCA's design, implementation and use of monitoring data to adapt the project's interventions affected the results. There was limited project documentation available for review, and no project theory of change that could guide the assessment of the effects of the project. Yet, stakeholders who had been involved in CERCA were available in all countries. Thus, the evaluation lead decided to engage CERCA partners as evaluators allowing the evaluation team access to the missing historical and contextual understanding of project implementation in the three countries.

With former CERCA staff on the evaluation team, it was possible to undertake a participatory development of CERCA's theory of change based on their knowledge of implementation and change pathways to achieve expected outcomes. It also became apparent that partners understood the project's implementation process and outcomes/impact quite differently, leading to the PPE taking this into account in their interpretation of the impact assessment.

*Source*: Ivanova *et al.* (2016)

### Managing a lack of data to assess change in rigorous ways

Most evaluations use a mix of primary and secondary data to assess change. Both pose particular challenges in PPE contexts. We highlight two issues here; others are found in the Guidance.

- Typical evaluation collects primary data from project beneficiaries, implementing staff and other stakeholders in real-time and observable implementation conditions to determine change. Yet, once a project's operational structure is disbanded—participants and staff have moved on with their lives—finding respondents, ensuring representativeness and managing recall data are critical concerns. Additionally, even if the adolescent participants can be identified, evaluators need to account that they will now be older, more developmentally mature and in a different stage of their lives, for example in education or in relationships. for their maturation.
- Secondary data reviews rely on the availability of past project evaluations and other studies and implementation documentation, which are often unavailable. Even if available, existing documentation may not be amenable to PPE outcome analysis and comparison of change.

The case example below highlights the creative use of existing secondary data and realistic-yet-feasible primary data collection to answer PPE evaluation questions.

Re-purposing existing project data for post-project evaluation: The Baylor Pediatric AIDS Initiative in the United Republic of Tanzania

From 2008 to 2013, Baylor University implemented a 7-year research project in the United Republic of Tanzania to strengthen services for prevention, early detection, treatment and care of paediatric HIV/AIDS in the Southern Highlands and the Lake zones.

A mixed-method post-project evaluation was carried out to assess the extent that the project's goals and objectives were achieved and to provide guidance and lessons learned for future projects, in particular related to effectiveness and sustainability. Geographic access and time and budget limitations for primary data collection were significant challenges, necessitating new ways to approach data collection.

The evaluation team had access to ample documentation and databases from the project period. It determined that a quantitative evaluation strategy could use the existing data for the aims of the PPE evaluation. Samples for quantitative data analysis, including a comparison group, were derived from databases of activities from existing large population samples of the Baylor International Pediatric AIDS Initiative that allowed evaluators to analyse data in the way they wanted. As the sample was not population-based, using the existing data introduced some bias but negated the need for new quantitative data collection.

To supplement, complement and verify quantitative analyses, primary qualitative data were collected through key informant interviews, focus group discussions and observations. It was not possible to draw a systematic or random sample of past participants, and instead, the project purposively sampled a subset of communities, facilities and beneficiaries.

*Source*: Bernstein *et al.* (2015)

### Assessing context, complexity and sustainability effects

Project documentation tends to lack information on contextual factors that help explain the nature and direction of change, such as the presence of other projects operating in the same area that could have influenced project trajectories and outcomes. This is compounded in PPE, as such information needs to be identified retrospectively. Reconstruction of external events may be as simple as interviews and historical policy analyses (case example below). Relatively new evaluation methods, such as Contribution Analysis and QUIP discussed in the Guidance, offer opportunities to understand complexity, contribution, sustainability and unanticipated or emerging outcomes.

Gaining an understanding of context to explain project outcomes: the strengthening collective response of the government to end child marriage through a district-level convergence approach in India

The MAMTA-Health Institute for Mother and Child, New Delhi, in partnership with district administrations, undertook a 3-year project (2012–15) to support cross-departmental convergence of government efforts to end child marriage in Sawai Madhopur, Rajasthan, and Jamui, Bihar. In the context of the evaluation, it was essential to understand external factors, during project implementation and in the time between project closing and the post-project evaluation, that could have helped or hindered project efforts to improve multi-sectoral coordination.

The evaluation team worked with former project staff and current senior management of MAMTA to define retrospectively contextual factors at government, programme and community levels and in project implementation that might have influenced the project’s outcomes and their sustainability. They created a timeline of external and internal events, such as changes in child marriage policies at national and state levels. They identified an overlap of MAMTA's activities with a similar state-sponsored programme to end child marriage (in Rajasthan) that began mid-project and worked across different departments. This information led to new evaluation questions about these contextual factors, and the team subsequently supplemented and confirmed information about these events through a review of policy documents and stakeholder interviews. The reconstructed context was critical to interpreting the significant differences in programme outcomes in the two districts.

*Source*: Chandra-Mouli *et al.* (2018)

PPE is exceptionally well suited to assess sustainability in real-time and to document unanticipated outcomes or emerging outcomes (i.e. results of project efforts that continue to evolve post-project due to participants’ and partners' own continued actions). Yet the review indicated that sustainability outcomes are often not well defined in former project documents. PPE evaluators should expect unanticipated and emerging effects and, with evaluation stakeholders, determine the extent to expand the PPE frame to document learnings useful for future projects.

## Discussion and a proposed way forward

The review identified a range of PPE challenges, both contextual and methodological. Those who engage with PPE need to anticipate such challenges from the beginning and be ready and willing to adjust the initial evaluation plan. While most methods used for PPE are the same as for other types of evaluation, their application may need to be adjusted to reflect the constraints of PPE conditions. On the other hand, PPE affords new opportunities to assess the sustainability of effects and identify unanticipated or emerging outcomes that only occur after projects have ended.

In the absence of well-thought-out plans for evaluation and consequently evidence-based conclusions, consciously designed and implemented PPE can and should play a more significant role in addressing such gaps, particularly for promising projects demonstrating innovation. There are a few possible reasons that PPE is so rarely done and, looking forward, a few possible actions that funding agencies, project implementers and researchers and evaluators could take in response.

The notable lack of PPE in the field is partly structural; funding agencies that support health and development programming do not typically request this type of evaluation given their project funding cycles. Even when PPE has been carried out, there is a gap in the accessibility of PPE reports. Beyond structural reasons, though, there appears also to be a reluctance to conduct PPE. Because PPE often does not lend itself to the most rigorous experimental design, many methodological purists and funding agencies believe it is not worth the investment.

This has led to a significant gap and lost opportunity to learn from the diversity of projects that currently exists. In the case of ASRH, a still-emerging field of practice in lower- and middle-income countries, projects are not often designed with evaluation in mind. Thus, an underappreciated and underexplored evaluation and learning opportunity exists to assess and document implementation processes and project outcomes and impact, and their sustainability. The WHO Guidance responds to this considerable gap.

The review indicates that PPE is possible, can be rigorous, and is valuable when more traditional evaluations have not been planned or carried out. PPE should be done more frequently, not only for ASRH projects but also for other health and development efforts. The Guidance proposes a way forward: (1) Create spaces and funding for such evaluations. Researchers and evaluators, funding agencies and project implementers should consider the utility of PPE. (2) Create demand for PPE. In the absence of a planned evaluation, funding agencies and governments that support the testing of programme innovations should request PPE when a particular innovation shows promise but lacks sufficient documentation. (3) Build and facilitate access to evidence of PPE methodological and contextual challenges and solutions. Most evaluation reporting remains in the grey literature and posting evaluation reports on existing government/organizational websites and evaluation clearinghouses will greatly aid in compiling experiences and lessons learned.
